# Omentin as a novel biomarker of metabolic risk factors

**DOI:** 10.1186/1758-5996-4-37

**Published:** 2012-07-26

**Authors:** Rei Shibata, Noriyuki Ouchi, Ryotaro Takahashi, Yuya Terakura, Koji Ohashi, Nobuo Ikeda, Akiko Higuchi, Hiroko Terasaki, Shinji Kihara, Toyoaki Murohara

**Affiliations:** 1 Department of Cardiology, Nagoya University Graduate School of Medicine, Nagoya, Japan; 2 Department of Molecular Cardiology, Nagoya University Graduate School of Medicine, 65 Tsurumai, Showa-Ku, Nagoya, 466-8550, Japan; 3 Department of Cardiology, Chunichi Hospital, Nagoya, Japan; 4 Department of Ophthalmology, Nagoya University Graduate School of Medicine, Nagoya, Japan; 5 Department of Biomedical Informatics, Osaka University Graduate School of Medicine, Nagoya, Japan

**Keywords:** Omentin, Adipocytokine, Metabolic disorders, Risk factors, Biomarkers

## Abstract

**Background:**

Omentin is an adipocytokine that is abundantly expressed in visceral fat tissue. We investigated the association of omentin with the number of metabolic risk factors.

**Finding:**

The study population comprised 201 Japanese men who underwent annual health checkups. Plasma omentin levels were determined by enzyme-linked immunosorbent assay. We divided the subjects into 4 groups according to omentin levels. A reduction of plasma omentin levels significantly correlated with an increase in the mean number of metabolic risk factors such as increased waist circumference, dyslipidemia, high blood pressure and glucose intolerance.

**Conclusions:**

Circulating omentin levels negatively correlated with the multiplicity of metabolic risk factors, suggesting that omentin acts as a biomarker of metabolic disorders.

## Findings

Obesity has become a major health problem in industrial countries with increasing prevalence in adults and children [1]. Obesity, in particular, excess of visceral adipose tissue, is causally linked with a cluster of metabolic disorders including glucose intolerance, dyslipidemia, and hypertension, also known as metabolic syndrome [2-4].

Accumulating evidence indicates that adipose tissue is an active endocrine organ that produces various bioactive substances, also known as adipocytokines or adipokines [4,5]. Excess adipose mass observed in obese individuals is linked with increased production of many adipocytokines including tumor necrosis factor-α and interleukin-6, which potentially promotes metabolic dysfunction [5,6]. Fat tissues also produce a smaller number of adipocytokines including adiponectin and Sfrp5, which are beneficial in the setting of obesity-linked complications [5,7,8]. The imbalance of production of these adipocytokines may cause the development of obesity-related metabolic and vascular disorders.

Omentin/intelectin-1 is an adipocytokine that exists in human blood stream [9-11]. While omentin is highly expressed in human visceral fat tissue, circulating omentin levels are reduced in obese subjects [12]. Omentin is also down-regulated in association with obesity-linked metabolic disorders including insulin resistance, glucose intolerance and type 2 diabetes [12-14]. However, nothing is known about the relationship between omentin and metabolic risk factors. Here, we examined whether circulating omentin levels associate with the multiplicity of metabolic risk factors.

This study included 201 Japanese men who visited Chunichi Hospital in Nagoya for an annual health checkup. All subjects had no history of cardiovascular disease and took no medication. All subjects enrolled in this study provided written informed consent. This study was approved by the ethics committee of the Nagoya University School of Medicine and the Chunichi Hospital.

We assessed the presence or absence of four metabolic risk factors: increased waist circumference, dyslipidemia, elevated blood pressure (BP), and dysglycemia/impaired glucose tolerance. An increase in waist circumference was defined as ≥85 cm. Dyslipidemia represented elevated triglycerides (>150 mg/dl) and/or low HDL cholesterol (<40 mg/dl). Elevated BP was defined as systolic BP of ≥ 130 mmHg or diastolic BP of ≥ 85 mmHg on repeated measurements. After an appropriate rest of 10minutes, sitting BP was measured. Dysglycemia/impaired glucose tolerance represented hyperglycemia (fasting glucose level of ≥110 mg/dl).

Venous blood samples were obtained for chemical analysis after an overnight fast. Plasma omentin levels were determined with omentin enzyme-linked immunosorbent assay (ELISA) kit (Bio Vendor, NC, USA). Heparin was used for plasma sampling for measurement of omentin. The intra-assay and interassay coefficients of variation of this kit were 4.1% and 4.8%, respectively. Standard assays were used to measure glucose, hemoglobin A1c, insulin, total cholesterol, HDL cholesterol, LDL cholesterol, triglycerides, creatinine and high-sensitive C-reactive protein (hsCRP) levels.

Summary statistics are presented as mean ± standard error (S.E.) for continuous variables. Non-parametric Kruskal-Wallis analysis of variance test and multiple logistic regression analyses were used to analyze the relationship between the number of metabolic risk factors and omentin levels. A value of p <0.05 was considered significant. All analyses were performed using JMP (version 6.03; SAS Institute Inc).

The study population compromised 201 Japanese men who underwent annual health checkups. Clinical characteristics are shown in Table 1. Mean values of BMI (body mass index), waist circumference, blood pressure, hemoglobin A1c, total cholesterol, LDL cholesterol, HDL cholesterol, triglyceride, and creatinine were within the normal range. Because the distributions of omentin were skewed, we used logarithmically transformed values for statistical analysis. The distributions of log-omentin levels have been confirmed to fit the normal distribution by Shapiro–Wilk tests (p < 0.01). Mean plasma log-omentin level was 2.66 ± 0.01 ng/ml.

**Table 1:** Clinical characteristics

**Parameters**	**Subjects (n = 201)**
Age (years)	53.9 ± 9.0
BMI (kg/m^2^)	23.3 ± 2.6
Waist circumference (cm)	84.7 ± 6.8
Systolic BP (mmHg)	116.6 ± 17.6
Diastolic BP (mmHg)	72.9 ± 12.1
Fasting glucose (mg/dl)	103.6 ± 12.4
Hemoglobin A1c (%)	5.06 ± 0.53
Fasting insulin (μIU/ml)	5.82 ± 3.8
Total cholesterol (mg/dl)	204.0 ± 34.8
LDL cholesterol (mg/dl)	110.7 ± 25.8
HDL cholesterol (mg/dl)	55.3 ± 13.8
Triglyceride (mg/dl)	137.2 ± 75.6
Creatinine (mg/dl)	0.87 ± 0.13
Log hsCRP (mg/dl)	2.63 ± 0.44
Log omentin (ng/ml)	2.66 ± 0.01

Data are presented as means ± S.E

**Figure 1:**
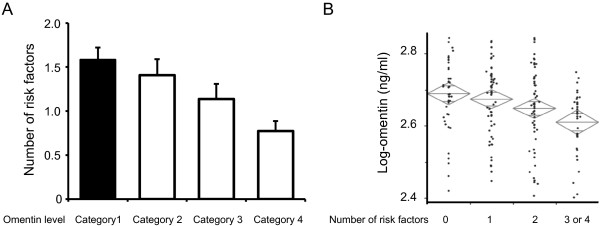
**Association between plasma omentin levels and number of metabolic risk factors.** Plasma omentin concentrations were determined by an ELISA kit. (A) Subjects were divided into 4 categories according to plasma log omentin concentration as follows: category 1, <2.59 ng/ml; category 2, ≥2.59 ng/ml, <2.66 ng/ml; category 3, ≥2.66 ng/ml, <2.72 ng/ml; and category 4, ≥2.72 ng/ml. (B) Log-omentin levels were divided into 4 categories according to 0, 1, 2, 3 or 4 metabolic risk factors.

We categorized the subjects into 4 groups according to plasma omentin levels as follows: category 1, <2.59 ng/ml; category 2, ≥2.59 ng/ml, <2.66 ng/ml; category 3, ≥2.66 ng/ml, <2.72 ng/ml; and category 4, ≥2.72 ng/ml. The number of subjects in the 4 categories were 50, 51, 49 and 51, respectively. The average number of metabolic risk factors significantly increased with the decline in the quartiles of plasma omentin levels (category 1: 1.58 ± 0.18; category 2: 1.40 ± 0.16; category 3: 0.98 ± 0.15; category 4: 0.78 ± 0.12) (P < 0.001) (Figure 1A). Omentin levels were also significantly lower in subjects with one or more risk factors than in those without risk factors (P = 0.001) (Figure 1B). Multiple logistic regression analyses after adjusting for age revealed that omentin levels were associated with a significant decrease in the number of metabolic risk factors (Table 2).

**Table 2:** Odds ratio of log-omentin levels for metabolic risk factors

**No. of metabolic risks**	**OR (95%CI)**	**P value**
0*	1.00 (-)	-
1	0.488 (-0.005, 0.003)	0.536
2	0.091 (-0.008, -0.004)	0.033
3-4	0.017 (-0.013, -0.004)	P < 0.001

Multiple logistic regression analyses include age and omentin

*Standard category. OR; odds ratio, CI: confidence intervals

In the present study, we, for the first time, demonstrate that circulating levels of omentin inversely correlate with the number of metabolic risk factors. Individuals with excess of visceral fat accumulation have a high risk of the development of metabolic syndrome [3]. Among various human tissues, visceral adipose tissue produces a large amount of omentin, and its gene expression in visceral fat depot is reduced in obese subjects [12]. Low levels of circulating omentin are associated with obesity-induced metabolic dysfunction such as insulin resistance and glucose intolerance [12,13]. These observations suggest that reduced levels of omentin may be an indicator of visceral fat accumulation, thereby correlating with the clustering of metabolic disorders.

Plasma concentration of omentin is associated with endothelium-dependent vasodilation [15]. Circulating omentin levels are negatively correlated with carotid intima-media thickness, which is a marker of early atherosclerosis [16]. Low levels of circulating omentin are also associated with the prevalence of coronary artery disease [17,18]. These data suggest that omentin may represent a biomarker for not only metabolic disorders, but also cardiovascular diseases.

The role of omentin in regulating metabolic function is poorly defined. A previous study showed that omentin stimulates glucose uptake in response to insulin in cultured adipocytes [9], suggesting that omentin exerts beneficial actions on insulin sensitivity. Future experimental researches are required to clarify the functional significance of omentin in the context of metabolic diseases.

In conclusion, the present study indicates that lower concentration of circulating omentin is linked with an increase in multiplicity of metabolic risk factors, suggesting that omentin serves as a biomarker for assessment of metabolic risk factors.

## Abbreviations

BMI, body mass index; BP, blood pressure; ELISA, enzyme-linked immunosorbent assay; hsCRP, high-sensitive C-reactive protein.

## Competing interests

The authors declare that they have no competing interests

## Author contributions

R.S., N.O., R.T., K.O., and T.M. designed and carried out the studies. N.I., A.H., T.Y., H. T., and S.K. analyzed the data. N.O. and R.S. wrote the paper. All authors read and approved the final manuscript.

## References

[B1] Flegal KM Carroll MD Kuczmarski RJ Johnson CL Overweight and obesity in the united states: Prevalence and trends, 1960-1994 Int J Obes Relat Metab Disord 1998 22 39 47 10.1038/sj.ijo.0800541 9481598

[B2] Nielsen S Jensen MD Obesity and cardiovascular disease: Is body structure a factor? Curr Opin Lipidol 1997 8 200 204 10.1097/00041433-199708000-00002 9253535

[B3] Despres JP Lemieux I Abdominal obesity and metabolic syndrome Nature 2006 444 881 887 10.1038/nature05488 17167477

[B4] Matsuzawa Y Therapy insight: Adipocytokines in metabolic syndrome and related cardiovascular disease Nat Clin Pract Cardiovasc Med 2006 3 35 42 10.1038/ncpcardio0380 16391616

[B5] Ouchi N Parker JL Lugus JJ Walsh K Adipokines in inflammation and metabolic disease Nat Rev Immunol 2011 11 85 97 10.1038/nri2921 21252989 PMC3518031

[B6] Ouchi N Kihara S Funahashi T Matsuzawa Y Walsh K Obesity, adiponectin and vascular inflammatory disease Curr Opin Lipidol 2003 14 561 566 10.1097/00041433-200312000-00003 14624132

[B7] Shibata R Ouchi N Murohara T Adiponectin and cardiovascular disease Circ J 2009 73 608 614 10.1253/circj.CJ-09-0057 19261992

[B8] Walsh K Adipokines, myokines and cardiovascular disease Circ J 2009 73 13 18 10.1253/circj.CJ-08-0961 19043226

[B9] Yang RZ Lee MJ Hu H Pray J Wu HB Hansen BC Shuldiner AR Fried SK McLenithan JC Gong DW Identification of omentin as a novel depot-specific adipokine in human adipose tissue: Possible role in modulating insulin action Am J Physiol Endocrinol Metab 2006 290 E1253 E1261 10.1152/ajpendo.00572.2004 16531507

[B10] Schaffler A Neumeier M Herfarth H Furst A Scholmerich J Buchler C Genomic structure of human omentin, a new adipocytokine expressed in omental adipose tissue Biochim Biophys Acta 2005 1732 96 102 10.1016/j.bbaexp.2005.11.005 16386808

[B11] Tsuji S Uehori J Matsumoto M Suzuki Y Matsuhisa A Toyoshima K Seya T Human intelectin is a novel soluble lectin that recognizes galactofuranose in carbohydrate chains of bacterial cell wall J Biol Chem 2001 276 23456 23463 10.1074/jbc.M103162200 11313366

[B12] de Souza Batista CM Yang RZ Lee MJ Glynn NM Yu DZ Pray J Ndubuizu K Patil S Schwartz A Kligman M Fried SK Gong DW Shuldiner AR Pollin TI McLenithan JC Omentin plasma levels and gene expression are decreased in obesity Diabetes 2007 56 1655 1661 10.2337/db06-1506 17329619

[B13] Pan HY Guo L Li Q Changes of serum omentin-1 levels in normal subjects and in patients with impaired glucose regulation and with newly diagnosed and untreated type 2 diabetes Diabetes Res Clin Pract 2010 88 29 33 10.1016/j.diabres.2010.01.013 20129687

[B14] Tan BK Adya R Farhatullah S Lewandowski KC O'Hare P Lehnert H Randeva HS Omentin-1, a novel adipokine, is decreased in overweight insulin-resistant women with polycystic ovary syndrome: Ex vivo and in vivo regulation of omentin-1 by insulin and glucose Diabetes 2008 57 801 808 10.2337/db07-0990 18174521

[B15] Yamawaki H Tsubaki N Mukohda M Okada M Hara Y Omentin, a novel adipokine, induces vasodilation in rat isolated blood vessels Biochem Biophys Res Commun 2010 393 668 672 10.1016/j.bbrc.2010.02.053 20170632

[B16] Shibata R Takahashi R Kataoka Y Ohashi K Ikeda N Kihara S Murohara T Ouchi N Association of a fat-derived plasma protein omentin with carotid artery intima-media thickness in apparently healthy men Hypertens Res 2011 34 1309 1312 10.1038/hr.2011.130 21814208

[B17] Zhong X Zhang HY Tan H Zhou Y Liu FL Chen FQ Shang DY Association of serum omentin-1 levels with coronary artery disease Acta Pharmacol Sin 2011 32 873 878 10.1038/aps.2011.26 21602837 PMC4003121

[B18] Shibata R Ouchi N Kikuchi R Takahashi R Takeshita K Kataoka Y Ohashi K Ikeda N Kihara S Murohara T Circulating omentin is associated with coronary artery disease in men Atherosclerosis 2011 219 2 811 4 10.1016/j.atherosclerosis.2011.08.017 21925659

